# Food Consumption and Dietary Patterns of Local Adults Living on the Tibetan Plateau: Results from 14 Countries along the Yarlung Tsangpo River

**DOI:** 10.3390/nu13072444

**Published:** 2021-07-17

**Authors:** Chenni Zhou, Mo Li, Lu Liu, Fangjie Zhao, Wenfeng Cong, Fusuo Zhang

**Affiliations:** 1 Key Laboratory of Plant-Soil Interactions, Ministry of Education, College of Resources and Environmental Sciences, National Academy of Agriculture Green Development, China Agricultural University, Beijing 100193, China; chenni2018@126.com (C.Z.); limo_0125@163.com (M.L.); liulucau@foxmail.com (L.L.); zhangfs@cau.edu.cn (F.Z.); 2 Key Laboratory of Forest Ecology in Tibet Plateau, Ministry of Education, Institute of Tibet Plateau Ecology, National Forest Ecosystem Observation & Research Station of Nyingchi Tibet, Key Laboratory of Ecological Security in Tibet, Tibet Agriculture & Animal Husbandry University, Nyingchi 860000, China; 3 College of Resources and Environmental Science, Nanjing Agricultural University, Nanjing 210095, China; fangjie.zhao@njau.edu.cn

**Keywords:** food consumption, dietary intake, dietary patterns, PCA, Tibetan adults, Tibetan Plateau

## Abstract

The distinct Tibetan regional diet is strongly influenced by the regional biogeography, indigenous traditions, popular religious beliefs and food taboos. In the context of the nutritional transition in Tibet, studies seldom report on the food consumption and dietary patterns of Tibetan residents. This is a cross-section study of 552 local adults (≥18 years old, 277 men and 275 women) living in 14 agricultural countries along the Yarlung Tsangpo River. Dietary intakes were assessed by a culturally specific FFQ and compared with the Chinese Dietary Pagoda (2016). Dietary Patterns were extracted by using PCA method. The binary logistic regression model was applied to assess the association between independent variables (genders, regions and age groups) and adherence to dietary patterns. With the exception of meat (100 ± 260 g/day) and soybean nuts (42 ± 12 g/day), which exceeded the recommended dietary intakes of CDP, the dietary intake of other foods were not up to the recommended value. In particular, the intake of aquatic products (2 ± 0.1 g/day), vegetables (90 ± 19 g/day), dairy products (114 ± 29 g/day), cereals (117 ± 27 g/day) and fruits (97 ± 25 g/day) were seriously inadequate, which were 95%, 70%, 62%, 53.2% and 51.5% lower than the recommended intakes, respectively. Four dietary patterns were identified. “Local traditional diet” was characterized by a high intake of tsampa (roasted highland barley flour), culturally specific beverages (sweet tea and yak buttered tea), potato and yak beef and was associated with female, rural and older adults (≥51 years old). The male, urban and 18~30 years old group had a higher adherence score with the “Han diet”, which was comprised of rice, pork, dumplings, eggs, milk and cabbage. The “Beverage diet”, which mainly include tsampa, chang (homemade barley wine) and sweet tea, was associated with the following group: female, urban and aged 18~30 years. The “Out-sourced diet” pattern, consisting of mainly rice, steam bread and some processed meat, was associated with being male, urban and 18–30 years of age. These findings indicate that the dietary practice of the Tibetan people still has strong local characteristics, but it is also undergoing a dietary transition with the penetration of the Chinese Han diet and the increased consumption of outsourced (processed) foods. The unbalanced dietary intake of Tibetan residents should be taken seriously by all parties.

## 1. Introduction

Numerous epidemiological studies provide evidence that there is a definite association between regional chronic disease and dietary patterns [1,2]. Therefore, increased attention has been paid to the rational dietary structure and balanced nutrient intake. Food consumption surveys are fundamental to the analysis of food security, the assessment of diet and nutrient intake and the developing nutrition interventions [3,4]. The food frequency questionnaire (FFQ) is one of the most commonly used assessment tools for diet assessment, which is a better measurement than those obtained using three days weighting method or 24 h dietary recall [2] and has been extensively used in epidemiological studies [1,2,3]. Meanwhile, dietary patterns use the FFQ and food intake data to identify the type of foods that are significant for a disease [1,2] or a specific group of people [5,6]. Compared with the traditional analysis of single food or nutrients, dietary pattern analysis has been identified as a more comprehensive method to characterize diet [1] and it can offer a broader picture of food consumption, reflect a more realistic dietary habit and provide some practical approaches to disease prevention. According to previous studies, dietary pattern is also closely associated with sociodemographic, occupational factors and lifestyle factors [7,8]. Therefore, it is particularly important to investigate food consumption in different genders, age groups and regions. Moreover, in rural areas, food accessibility is not as diverse as in urban and sub-urban areas. It is of great significance to carry out the survey of food consumption and food source in remote areas to balance the dietary gap between different regions.

In China, although the food consumption pattern is changing and the nutrition status of residents is also greatly improved, the research on the dietary nutrition characteristics of residents mainly focuses on the economically developed eastern regions and the urbanization areas [9,10]. However, for the vast territory of China, the dietary gap between regions is still obvious. The dietary data of the residents in rural and remote areas of China is still extremely lacking, especially for the economically underdeveloped remote mountainous areas in western China [11]. The national nutrition problem and the concern to realize nutrition balance has become an urgent problem that needs to be solved in China. Therefore, in order to carry out effective nutrition intervention, it is crucial to understand the food consumption and dietary pattern in different regions of China.

In the Tibet Autonomous region, most areas are not suitable for grain production and more calories and nutrients may be needed to maintain the basic metabolism of the human body on the plateau [12]. The Tibetan distinct regional diet is strongly influenced by the regional biogeography, indigenous traditions, popular religious beliefs and food taboos. Local traditional foods occupy an important place in the diet [13]. However, this is changing with the improvement of marketization, the multiple influences that economic development processes may have had on local diets, which include the introduction of new income sources and employment opportunities, infrastructural and transportation expansion, as well as environmental change [14]. Studies have shown that with the increase in purchased rice and wheat flour, the proportion of highland barley consumption of rural residents in Tibet has gradually decreased from 70% in the early 1990s to 50% in 2015 [15,16]. Nevertheless, although the food consumption structure of Tibetan residents is developing in a diversified direction, the traditional diet of local residents has also been enriched under the background of market opening and little research has been conducted on the food consumption characteristics and dietary pattern of local residents in Tibet. However, understanding the dietary practices of Tibetan adults may help target interventions to improve diet and nutrient status of residents living in the alpine regions of western China. Given the dearth of information on the subject, we undertook the present study with the objective of assessing the daily intakes of local residents in Tibet by comparing the recommended values of the Chinese Dietary Pagoda (2016), extracting the main dietary patterns of Tibetan residents of different genders, regions and age groups.

## 2. Materials and Methods

### 2.1. Study Design

**Figure 1:**
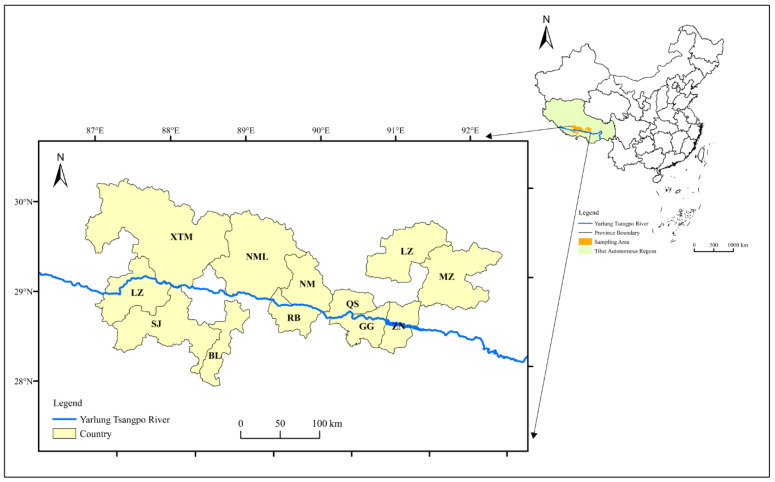
Distribution of sample countries.

**Figure 2:**
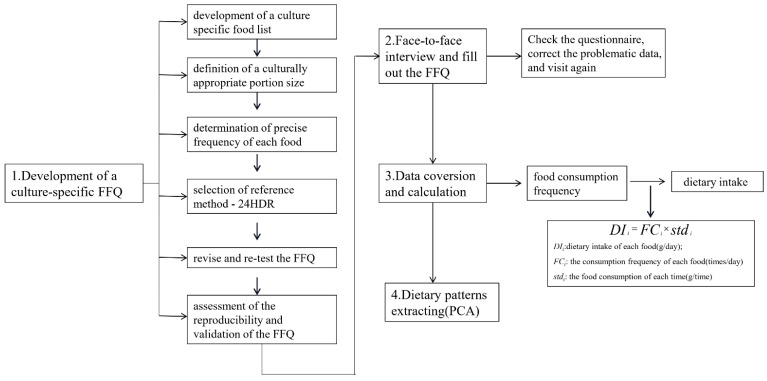
Methodological design for (1) the development of FFQ; (2) interview and fill out the FFQ; (3) data conversion and calculation; (4) dietary patterns extracting FFQ: food frequency questionnaire; 24 HDR: 24 h dietary recall.

For this study, 552 local Tibetan residents (≥18 years old) from 14 major agricultural countries (MedroGongkar (MZ), Chushur (QS), Nyemo (NM), Lhundup (LZH), Danang (ZN), Gonggar (GG), Sangzhuzi (SZZ), Namling (NML), Gyantse (JZ), Sakya (SJ), Lhatse (LZ), Thongmon (XTM), Panam (BL) and Rinpun (RB)) along the Yarlung Tsangpo River were quizzed about their eating habits. The distribution of sample sites is presented in Figure 1. All the participants’ dietary data were collected using a cultural-specific food frequency questionnaire that contained all local Tibetan foods, such as the following: tsampa (flour obtained from roasted highland barley grains), Tibetan sweet tea (Local black tea mixed with sweetened milk powder), yak buttered tea (salt cream tea), yak meat and chang (wine made from highland barley in a unique local manner). For each country, 20 urban residents and 20 rural residents were selected by simple random sampling and different genders were also considered. All participants were divided into three age groups (L_18~30 years old; M_31~50 years old; H_>50 years old) and interviewed face-to-face by trained professional interviewers during August 2020. The methodological design of the present study is schematically outlined in Figure 2.

Validated FFQs are a gold standard method of collecting information on quantity and frequency of foods consumed retrospectively [17]. We conducted a pre-survey to develop a draft food list in order to complete a culturally-specific FFQ. Considering the relative singularity of dietary structure and ethnic uniqueness of Tibetan local residents, we first verified the validity of the FFQ by using a 24 H dietary record in Lhundup County before applying it in the study. The FFQ was revised twice by local nutritional staff and its re-tested results ensure that this cultural-specific FFQ can fit local daily food consumption patterns well. The final version of the FFQ included 165 food items with an average standard portion size, proper frequency categories and food sources (self-produced or outsourcing).

### 2.2. Food Consumption Database

We input the original FFQ into the computer, including food items and the frequency of consumption and recorded volumes according to the standard portion size. The intake amount and the portion size were explained to each participant by the standard catalog of pictures of each kind of food, which were edited by NINH of CCDC and used in CNNHS [18]. The daily intake amount of each item of food was calculated according to the food intake amount for each time and intake frequency.

Since the Chinese Diet Pagoda (CDP) is the authoritative dietary guide for the Chinese people [19], we divided 165 food items into 11 food categories according to the CDP (2016 version) and covered “grains and potatoes”, “vegetables”, “fruits”, “meats”, “aquatic products”, “eggs”, “dairy products”, “soybeans and nuts”, “edible oil” and "salt”. Then, the dietary intake of each food category was calculated.

### 2.3. Statistic Analysis

A *p*-value lower than 0.05 was predetermined in all hypothesis testing procedures. The Shapiro–Wilk test was used to determine whether all the dietary intakes had a normal distribution. Mean, standard deviation, median and percentiles of distribution of dietary intakes were calculated and compared by analysis of variance (ANOVA) with gender (2 levels), region (2 levels) and age (3 levels). Dietary intakes were assessed based on the CDP (2016 version), which is the national standard for Chinese people. Reference values from CDP were the following: “cereals” at 250 g; “vegetables” at 300 g; “fruits” at 200 g; “meats” at 40 g, “aquatic products” at 40 g, “eggs” at 40 g, “dairy products” at 300 g, “soybeans and nuts” at 25 g, “oil” at 25 g, “salt” at 6 g and “water” 1500 mL. One sample *t*-tests were applied to compare the means of dietary intakes against reference values to assess the risk of adequate intakes.

Principal component analysis (PCA) was used to identify the dietary patterns by using R version 3.6.1 and the principal function in the psych package [20]. Dietary patterns were extracted from 105 food items collated from a 165 food items FFQ and 60 food items were removed for not constituting the eating habits of the analyzed population (consumption <5% of the sample) [7]. According to the eigenvalue (greater or equal to 1) of food intake, the first four dietary patterns were extracted. A total of 86.9% variance were explained from dietary pattern 1—Local traditional diet (37.3%); dietary pattern 2—Han diet (21.9%); dietary pattern 3—Beverage diet (13.9%); and dietary pattern 4—Out-sourced diet (12.6%) (Supplementary Figure S1). A factor loading ≥0.3 or ≤−0.3 was considered significant for this sample size [18]. A table of the food items and their factor loadings are attached (Supplementary Table S1). Varimax orthogonal rotation with Kaiser normalization was performed to reduce correlations between factors and to increase interpretability of the results. Kaiser–Meyer–Olkin (KMO) measure of sampling adequacy was 0.661 while Bartlett’s test of sphericity was significant (*p* < 0.001). Standardized dietary pattern scores were calculated for each participant for each dietary pattern using the regression method. The binary logistic regression model was applied to assess the association between independent variables (genders, regions and age groups) and adherence to dietary patterns (adherence below the median versus adherence above the median) [7].

## 3. Results

### 3.1. Demographic Characteristics of the Subjects

A total of 552 local Tibetan adults were included in the food survey with 277 (50.2%) men and 275 (49.8%) women, with an average age of 39 ± 14 years old. The region distribution was similar between urban (*n* = 280, 50.7%) and rural (*n* = 272, 49.3%) areas. All the subjects were divided into three age groups, which are 18~30 years old (*n* = 172, 30.6%), 31~50 years old (*n* = 230, 40.9%) and >51 years old (*n* = 160, 28.5%). Detailed information about subjects is provided in Supplementary Table S1.

### 3.2. Dietary Intakes of Local Tibetan Residents

The consumption of 11 kinds of food categories by Tibetan residents was as follows: with the exception of meat (100 ± 260 g/day) and soybean nuts (42 ± 12 g/day), which exceeded the recommended dietary intakes of CDP (more than 150% and 68%, respectively), the dietary intake of other foods were not up to the recommended value. In particular, the intakes of aquatic products (2 ± 0.1 g/day), vegetables (90 ± 19 g/day), dairy products (114 ± 29 g/day), cereals (117 ± 27 g/day) and fruits (97 ± 25 g/day) were seriously inadequate and were 95%, 70%, 62%, 53.2% and 51.5% lower than the recommended intakes, respectively. After a one sample *t*-test, there was a significant difference between the actual dietary intakes and the recommended intakes of all food categories (Table 1).

### 3.3. Dietary Intakes of Urban and Rural Tibetan Local Residents

**Figure 3:**
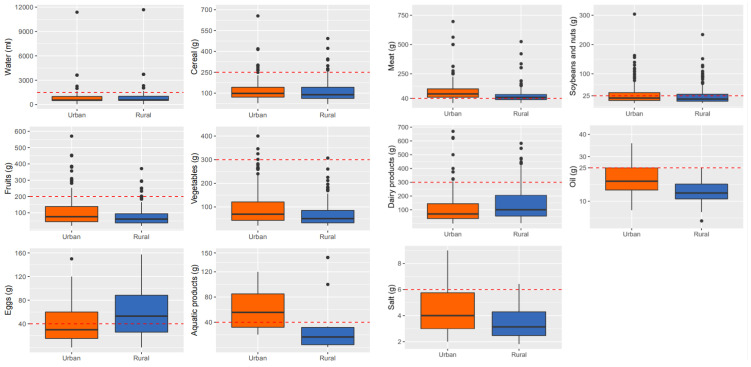
Dietary intakes of urban and rural Tibetan local residents.

Urban and rural residents have different dietary intakes of different food categories. According to the Chinese Diet Pagoda, with the exception of the dietary intakes of meat, eggs (for rural residents) and aquatic foods (for urban residents) which exceeded the recommended amount, the intakes of the other foods were insufficient (Figure 3). By comparing the dietary intake of urban and rural residents, we found that the dietary intakes of fruits, vegetables, meats, aquatic products, oil and salt of urban residents are significantly higher than that of rural residents (*p* < 0.01); however, the dietary intake of eggs and dairy products is significantly lower than that of rural residents (*p* < 0.01).

### 3.4. Dietary Intakes of Tibetan Local Residents of Different Genders

**Figure 4:**
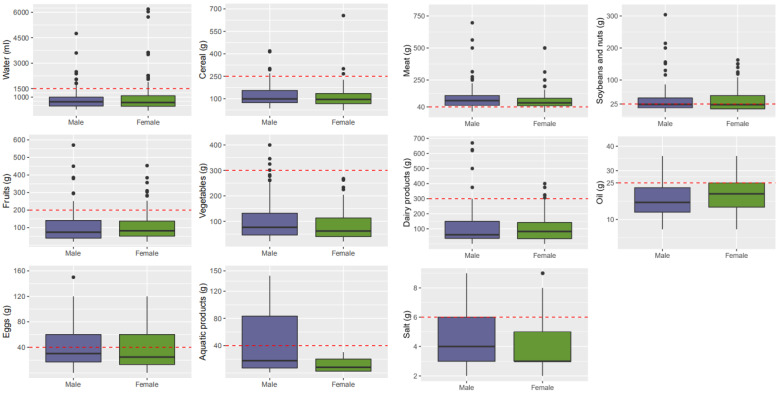
Dietary intakes of Tibetan local residents of different genders.

From the analysis of dietary intakes of different genders, we found that, with the exception of the intakes of meat, soybean and nuts for all genders which reached the recommended amount of the Chinese resident’s dietary pagoda, the intakes of other foods were insufficient (Figure 4). Men consumed more of all foods compared to women except for water and oil and, particularly, the intakes of vegetable, meats and salt by men were significantly higher than that of women (*p* < 0.01).

### 3.5. Dietary Intakes of Tibetan Local Residents in Different Age Groups

**Figure 5:**
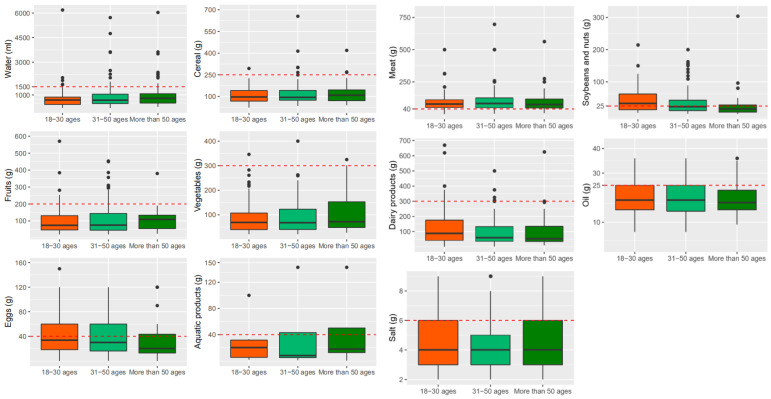
Dietary intakes of Tibetan local residents in different age groups.

Young adults (18~30 years old) consumed more eggs, meat, soybeans and nuts, dairy products, oil and salt than adults aged 31 to 50 and those aged >51 years old (Figure 5). The intakes of water by young adults (18~30 years old) and adults aged 31 to 50 were significantly lower than those aged >50 years old (*p* < 0.05); there was no significant difference between the two younger groups. The intakes of eggs and dairy products by young adults (18~30 years old) and adults aged 31 to 50 were significantly higher than those by the aged >50 years old (*p* < 0.05). Moreover, there was no significant difference between the two younger groups. The intakes of salt by young adults (18~30 years old) were significantly higher than the intakes of adults aged 31 to 50 and those aged >50 years old (*p* < 0.05); there was no significant difference between the two older groups.

### 3.6. Dietary Patterns of Tibetan Local Residents

**Figure 6:**
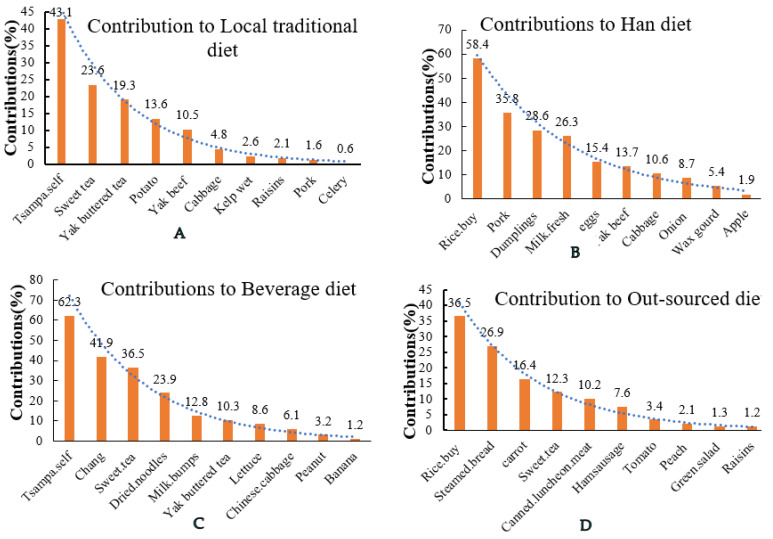
The 10 highest contributing food groups to each dietary pattern. P.S. Tsampa.self: home-grown tsampa; Rice.buy: rice purchased at the market. (**A**). The 10 highest contribution food groups to Local traditional diet. (**B**). The 10 highest contribution food groups to Han diet. (**C**). The 10 highest contribution food groups to Beverage diet. (**D**). The 10 highest contribution food groups to Out-sourced diet.

According to the preset criteria of variable selection (i.e., eigenvalue > 1 and the accumulation of total variance is 86.9%), there are four principal components; dietary patterns were retained by PCA method. Dietary patterns were named in accordance with the interpretability and characteristics of the items retained in each pattern and the items with the highest factor loadings were the ones that most influenced the interpretation and denomination of factors [17]. Food items with factor loadings ≥0.3 or ≤−0.3 was considered significant for this sample size (Supplementary Table S3) [2,8]. The contributions of food items (top 10 food items that contribute the most) for each dietary pattern are shown in Figure 6. The dietary pattern identified by principal component 1, which is labeled the Local traditional diet (the eigenvalue is 3.24, the variance contribution is 37.3% and the cumulative variance contribution is 37.3%), was characterized by a high intake of tsampa, culturally specific beverages (sweet tea and yak buttered tea), potato and yak beef. The dietary pattern identified by principal component 2, which is labeled Han diet (the eigenvalue is 1.9, the variance contribution is 21.9% and the cumulative variance contribution is 59.2%), is comprised of rice, pork, dumplings, eggs, milk and cabbage. The dietary pattern identified by principal component 3, which is labeled Beverage diet (the eigenvalue is 1.21, the variance contribution is 13.9% and the cumulative variance contribution is 74.3%), mainly includes tsampa, chang (homemade barley wine) and sweet tea. Dietary pattern identified by principal component 4, which is labeled Out-sourced diet (the eigenvalue is 1.09, the variance contribution is 12.6% and the cumulative variance contribution is 86.9%), mainly includes rice, steam bread and some processed meat, such as canned luncheon meat and ham-sausage.

For the first dietary pattern (local traditional diet), there were significant differences in the distribution among the different genders (*p* = 0.029), different regions (*p* < 0.001) and different age groups (*p* = 0.035) (Table 2). Associated with the greater adherence to the “local traditional diet” pattern was the group that included the following: female, rural and age group ≥51years. There were significant differences in the distribution of the second dietary pattern (Han diet) between regions (*p* < 0.001) and age groups (*p* < 0.001), but no significant differences between genders (*p* = 0.133). The male, urban and 18~30 years old group had a higher adherence score. For the Beverage diet, similar to the second diet, there were significant differences between genders (*p* = 0.011), regions (*p* = 0.046) and age groups (*p* = 0.027) and lower adherence to this dietary pattern was associated with the following group: female, urban and age 18~30 years. The distribution of the fourth dietary pattern (Outsourcing diet) was significantly different among genders (*p* = 0.0034), region (*p* < 0.001) and age groups (*p* < 0.001). In addition, the “Out-sourced diet” pattern was associated with being male, urban and 18–30 years of age.

## 4. Discussion

There are very limited published data on Tibetan dietary intakes and patterns and only a few studies were about specific subgroups such as Tibetan mothers [8,21] and children in rural areas [22]. To the best of our knowledge, the present study is the first comprehensive analysis and the most recent study of the dietary pattern of Tibetan adults that target different genders, regions and age groups. Based on the FFQ data from 14 countries, the results showed that the dietary intakes remain imbalanced. Compared with the dietary pagoda of Chinese residents (2016), the intake of other food categories did not reach the recommended intake except for meat, soybeans and nuts, which was not consistent with previous research [23]. Michael Dermience et al. found the consumption of meat was generally low across all counties in Tibet [22]. In the present study, the meat consumption of Tibetan residents is high (100 ± 26 g/day) and is greater than the recommended value 40~75 g/day, but the consumption structure is fairly monotonous as it mainly consists of yak beef and mutton pork is served as supplements. However, we found that with the development of infrastructure and the lack of awareness of the drawbacks of processed food among Tibetan residents, the consumption of processed meat products has increased significantly, which is also one of the reasons causing overall meat intake to exceed the standard (Figure 6). The intakes of aquatic products, vegetables, fruits and grains were the most deficient, which were 95%, 70%, 52.5% and 53.2% below the recommended value, respectively. The reason for the poor diversity of vegetables and fruit consumption probably lies in the harsh climate of the Himalayan plateau, which does not allow their cultivation [24], and in the lack of income, which prevents the rural people to buy imported foods. Intake of aquatic products is extremely low for religious and cultural reasons [22]. Although a few previous studies have only demonstrated the high frequency of grain consumption among Tibetans [22] and the fact that grain foods provide major nutrients [21], there exists few research on the adequacy of their grain intake. According to the results of a food consumption survey in 2010, the consumption of cereal in Tibet is 104.7 g/day/adult (highland barley), 60.7 g/day/adult (rice) and 76.8 g/day/adult (wheat flour), with an average intake of 80.7 g/day/adult [25]. In this study, tubers were also included in the staple food group, with an average intake of 117 ± 27 g/day/adult, and cereal contributed 87 ± 15 g/day/adult. During the 10 years, the staple food intake of local residents did not change but the total intake was insufficient.

Analyzing dietary patterns (rather than foods or nutrients) has been recommended as an effective method for studying diet and its relationship to health due to the complex interaction between different food components [26,27]. Dietary patterns can be derived using an a priori (hypothesis-driven) [28] or an a posteriori (data-driven) approach [18,29,30]. Although data-driven dietary patterns may not be as polarized as hypothesis-driven dietary patterns, they can more accurately reflect actual eating habits as combinations of food consumed in different groups of the population, rather than relying on current knowledge [18]. Data-driven dietary patterns are identified using statistical methods, for example, K-means cluster analysis, principal component analysis (PCA), explanatory factor analysis (EFA) and finite mixture modeling [6,8,26,31,32]. In this study, we used the method of PCA to determine the dietary patterns based on data from the 165 food items FFQ of 552 local residents and obtained four dietary patterns in the Tibet Autonomous Region, P.R. China, which were “local traditional diet”, “Han diet”, “Beverage diet” and “Outsourcing diet”. Similar to the present study, Cao et al. (2020), in a survey of 7555 adults from Jiangsu province, China, showed four dietary patterns in the Chinese Han population by using PCA; these include the “meat diet”, “Healthy diet”, “Traditional diet” and “Fried food with staple food” [2]. Cattafesta et al. (2020) identified three dietary patterns that include “local traditional”, “traditional Brazilian” and “industrialized” in a cross-sectional epidemiological study of 740 farmers in Brazil [7].

The results of this study cannot be directly compared with similar studies in other parts of China due to the uniqueness of Tibetan culture and religion. However, we can evaluate that the diet of Tibetan residents has the following characteristics: (1) Tibetans still rely more on traditional foods and locally produced food than outsourced food, which demonstrates that dietary practices are strongly influenced by the regional biogeography, indigenous traditions, popular religious beliefs and food taboos [4]. (2) Han diet is permeating and enriching the Tibetan traditional diet. (3) Tibetan is a nation that loves to drink, whether it is alcohol or beverage. The local homemade highland barley liquor is the main type of alcohol, while the tea with cultural characteristics (sweet tea and yak buttered tea) is the main type of beverage. (4) Industrialized foods (i.e., processed meat) has appeared in the Tibetan food consumption pattern. Unfortunately, we did not find a “healthy diet” pattern that was dominated by various fruits and vegetables [2,8], nor did we find a “high rich in protein” pattern that was dominated by meat, eggs and milk [18] and a “prudent” pattern that was dominated by soy foods intake [17]. By analyzing the distribution of four dietary patterns, we found that males had higher adherence scores than females in three other dietary patterns except the “local traditional diet” pattern. A recent study of the dietary patterns of Brazilian farmers showed that men were associated with transport using their own vehicle and worked only with temporary crops; they had more opportunities to go out to work and eat than females [1], which is consistent with the case of Tibet. On the other hand, there was no difference in the distribution of the second dietary pattern (Han diet) between male and female. This may indicate that although the diet of the Han people is changing the local diet pattern, its distribution is still relatively small in the population and not enough to replace the local traditional diet pattern. There were significant differences in the distribution of the four dietary patterns between urban and rural areas. In addition to the “local traditional” pattern, the other three dietary patterns have a higher adherence score with the urban group, which showed that rural residents were more inclined towards the local traditional diet. Due to the convenience of infrastructure and transportation, people living in urban areas have more access to more diverse foods [33]. Moreover, with higher per-capita revenue, urban families are able to buy more varieties of food and consume more food overall [7]. Lower adherence to the “Han diet” and “Out-sourced diet” pattern was associated with age ≥51 years, while the same age group had higher adherence with the “local traditional diet” pattern. Even so, we did not find other dietary patterns (i.e., “vegetable-based”, “Western” or “prudent”) with adherence to the older group referred in other studies with older adults [1,18,29]. The relationship between age and dietary patterns is controversial. A negative association with age in the “Varied dietary” patterns (based on vegetables, fruits and soy products) was reported by Wang et al. (2017) [22], whereas a positive association between age and a vegetable-fruit pattern was found by Tseng and De Vellis [34]. It is worth noting that young people are more inclined to the “Han diet” pattern and the “outsourcing diet” pattern. We also assessed the dependence of local residents on self-produced and outsourcing foods. Results showed that in terms of food categories, they have two source attributes of both purchased and self-produced, males are more dependent on self-produced food than females and older residents (>50 years) are more dependent on self-produced food compared to younger residents (31–50 years and 18–30 years) (Supplementary Figure S2). This phenomenon has also been found in other previous studies about populations experiencing a nutrition transition. For example, Popkin and Reardon (2018) have reported that younger people tended to consume more processed foods and less traditional foods compared to older people [35]. Tibetan young adults aged between 18 and 30 are more likely to be influenced by Chinese Han culture because they have more opportunities to contact the outside world, which in turn increases the likelihood of not eating at home and resulting in greater adherence to the “outsourcing diet” pattern. The emergence of industrialized foods (i.e., processed meat) may reflect the better accessibility to more diversified food products. However, we were also concerned about excessive intake without considering the poor nutritional value and richness in salt, which may have an even greater negative impact on populations with low incomes and low educational level such as rural Tibetans [22].

Compared with other studies using PCA to extract dietary patterns, the present study has the advantages of the well-established data-driven statistical methodology and its high total variance (86.9%) explained by the four dietary patterns. The total explanatory rates of variance variation in the aforementioned studies were 28.1% [17], 43.939% [2], 28% [8], 27.4% [28], 18% [18] and 23.8% [7], respectively. Limitations of this study include the insufficient information on participants, especially socioeconomic, occupational, behavior and lifestyle factors, which are associated with food consumption and therefore influence dietary patterns. Moreover, we have not yet evaluated nutrient intakes in each dietary pattern nor have we explored the relationship between dietary patterns and endemic diseases. Furthermore, we have not yet adjusted food intakes with energy intake, which may result in different findings especially for gender and age comparisons. Moreover, the dietary patterns were extracted based on FFQ and, by using factor-loading matrix without taking the outcome variable into consideration, this may result in dietary patterns being suboptimal. Further studies should be conducted to explore the reproducibility and stability of the dietary patterns.

## 5. Conclusions

The present study was the first to provide detailed data on dietary intake and extract dietary patterns of Tibetan residents. Compared with the dietary pagoda of Chinese residents (2016), the dietary intake of Tibetan residents was imbalanced. The food intake of aquatic products, vegetables, fruits and grains were extremely inadequate. Four dietary patterns were extracted by PCA and female, rural and old adults (≥51 years old) have higher adherence scores with “Local traditional diet” while male, urban and 18~30 years old group had higher adherence scores with the “Han diet”. Lower adherence to the “Beverage diet” was associated with the female, urban and age 18~30 years group. The “Out-sourced diet” pattern was associated with greater adherence to the male, urban and age 18~30 years group. In conclusion, the dietary practice of the Tibetan people still has strong local characteristics, but it is also undergoing a dietary transition with the penetration of the Chinese Han diet and increased consumption of outsourcing (processed) foods. With the development of Tibet’s economy and infrastructure, this transition is inevitable; the unbalanced dietary intake of Tibetan residents should be taken seriously by all parties.

## Figures and Tables

**Table 1:** Food consumption status of Tibetan local residents.

	Actual Dietary Intakes	Recommended Dietary Intakes	Gap (%) ^a^	One Sample *t*-Test (*p* Value)
Cereals (g/day)	117 ± 27	250–400	−53.2	0.0008
Vegetables (g/day)	90 ± 19	300–500	−70	<0.0001
Fruits (g/day)	97 ± 25	200–350	−51.5	<0.001
Meats (g/day)	100 ± 26	40–75	150	0.0261
Aquatic products (g/day)	2 ± 0.1	40–75	−95	<0.0001
Eggs (g/day)	38 ± 11	40–50	−5	0.0311
Dairy products (g/day)	114 ± 29	300	−62	0.0422
Soybeans and nuts (g/day)	42 ± 12	25–35	68	0.0005
Oil (g/day)	20 ± 5	25-30	−20	0.0066
Salt (g/day)	4 ± 0.9	<6	−33.3	0.0029
Water (mL/day)	889 ± 256	1500–1700	−40.7	0.0004

^a^: Actual dietary intake divided by the lower limit value of the recommended intake. Gap = Actual/Recommended (%) ^−1^.

**Table 2:** The adherence scores to dietary patterns according to sociodemographic ^b^.

Variables	Local Traditional Diet	Han Diet	Beverage Diet	Outsourcing Diet
Gender	*p* = 0.029 *	*p* = 0.133	*p* = 0.011 *	*p* = 0.0034 **
Male	−0.48 (−0.91–−0.03))	0.14 (–0.42–0.80)	−0.12 (−0.62–0.50)	0.38 (−0.04–1.19)
Female	−0.30 (−0.82–0.35)	−0.12 (−0.66–0.46)	−0.17 (−0.61–0.35)	−0.09 (−0.65–0.54)
Region	*p* < 0.001 ***	*p* < 0.001 ***	*p* = 0.046 *	*p* < 0.001 ***
Urban	−0.18 (−0.74–0.49)	0.38 (−0.40–1.13)	−0.06 (0.60–0.53)	0.03 (−0.56–0.71)
Rural	0.04 (−0.63–0.76)	−0.09 (−0.65–0.54)	−0.12 (−0.66–0.46)	−0.43 (−0.94–0.23)
Age	*p* =0.035 *	*p* < 0.001 ***	*p* = 0.027 *	*p* < 0.001 ***
18~30 years	−0.12 (−0.69–0.58)	0.08 (−0.47–0.84)	−0.22 (−0.92–0.41)	0.07 (−0.43–0.85)
31~50 years	−0.17 (−0.80–0.37)	0.05 (−0.53–0.76)	0.06 (−0.54–0.91)	0.02 (−0.64–0.51)
≥51 years	0.13 (−0.42–0.97)	−0.02 (−0.59–0.53)	0.02 (−0.64–0.51)	−0.27 (−0.78–0.38)

^b^: The data are presented by median (IQR25–IQR75). *: *p* < 0.05; **: *p* < 0.01; ***: *p* < 0.001

## Data Availability

Not applicable.

## Supplementary Materials

The following are available online at https://www.mdpi.com/article/10.3390/nu13072444/s1, Figure S1: Proportion of information retained by each principal component, Figure S2: The intake of endogenous foods and exogenous foods by Tibetan residents, Table S1: Demographic characteristics of the subjects, Table S2: Intakes for food groups for 24 hour DR with FFQ, Table S3: Factor loadings for main dietary patterns.
