# Supplementary material for: Food Consumption and Dietary Patterns of Local Adults Living on the Tibetan Plateau: Results from 14 Countries along the Yarlung Tsangpo River
Source: Nutrients. 2021 Jul 17;13(7):2444. doi: 10.3390/nu13072444 (PMC8308694; doi:10.3390/nu13072444)
Supplement: Supplementary file 1 [file nutrients-13-02444-s001.zip › nutrients-1277630-supplementary.pdf]

Supplementary Table S1. Demographic characteristics of the subjects

Country	Gender		Region		Age		
	female	male	urban	rural	L(18-30yrs)	M(31-50yrs)	H(≥51yrs)
Danang	19	13	20	12	8	14	10
Chushur	17	20	20	17	11	18	8
Gonggar	17	23	20	20	9	18	13
MedroGongkar	17	21	20	18	10	17	11
Lhundup	23	17	20	20	13	17	10
Sangzhuzi	19	21	20	20	13	13	14
Panam	20	20	20	20	14	17	9
Nyemo	20	21	20	21	14	12	15
Namling	20	20	20	20	14	17	9
Sakya	20	20	20	20	12	19	19
Lhatse	19	21	20	20	12	19	9
Gyantse	23	21	20	24	14	17	13
Panam	21	19	20	20	16	14	10
Thongmon	20	20	20	20	12	18	10
Total	275	277	280	272	172	230	160
Ratio(%)	49.8	50.2	50.7	49.3	30.6	40.9	28.5

Supplementary Table S2. Intakes for food groups for 24 hour DR with FFQ

	Food group intakes		Spearman correlation coefficients
	24-H DR	FFQ	24-H DR vs FFQ
Cereals(g/day)	109±21	117±27	0.72
Vegetables(g/day)	79±11	90±19	0.55
Fruits(g/day)	82±12	97±25	0.42
Meats(g/day)	91±17	100±26	0.70
Aquatic products(g/day)	0.9±0.01	2±0.1	0.31
Eggs(g/day)	32±9	38±11	0.38
Dairy products(g/day)	98±19	114±29	0.57
Soybeans and nuts(g/day)	30±7	42±12	0.29
Oil(g/day)	26±6	20±5	0.41
Salt(g/day)	7±1.1	4±0.9	0.36
Water(ml/day)	1055±271	889±256	0.62
Median			0.48

Food group intakes for 24-H DR and FFQ are means±SD

Spearman correlation coefficients ≥0.26（P<0.05）and ≥0.35（P<0.01）

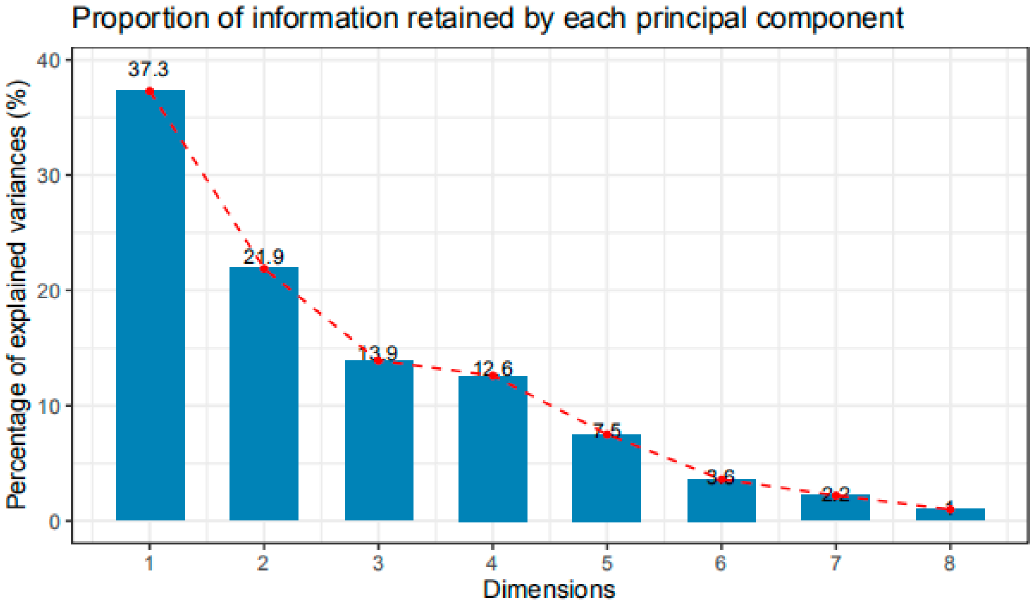

Supplementary Figure S1. Proportion of information retained by each principal component

Supplementary Table S3. Factor loadings for main dietary patterns [a]

**Local traditional diet**		**Han diet**		**Beverage diet**		**Out-sourced diet**	
**Foods**	**Factor loading**	**Foods**	**Factor loading**	**Foods**	**Factor loading**	**Foods**	**Factor loading**
Tsampa	0.633	Rice	0.796	Tsampa	0.531	Rice	0.549
Sweet tea	0.549	Pork	0.751	Chang	0.526	Steamed bread	0.496
Yak buttered tea	0.514	Dumplings	0.633	Sweet tea	0.463	carrot	0.427
Potato	0.422	Milk.fresh	0.591	Dried noodles	0.421	Sweet tea	0.377
Yak beef	0.408	eggs	0.420	Milk.bumps	0.364	Canned luncheon meat	0.341
Cabbage	0.365	Yak beef	0.328	Yak buttered tea	0.322	Hamsausage	0.309

a: the absolute value of food items factor loadings ≥0.3 are presented

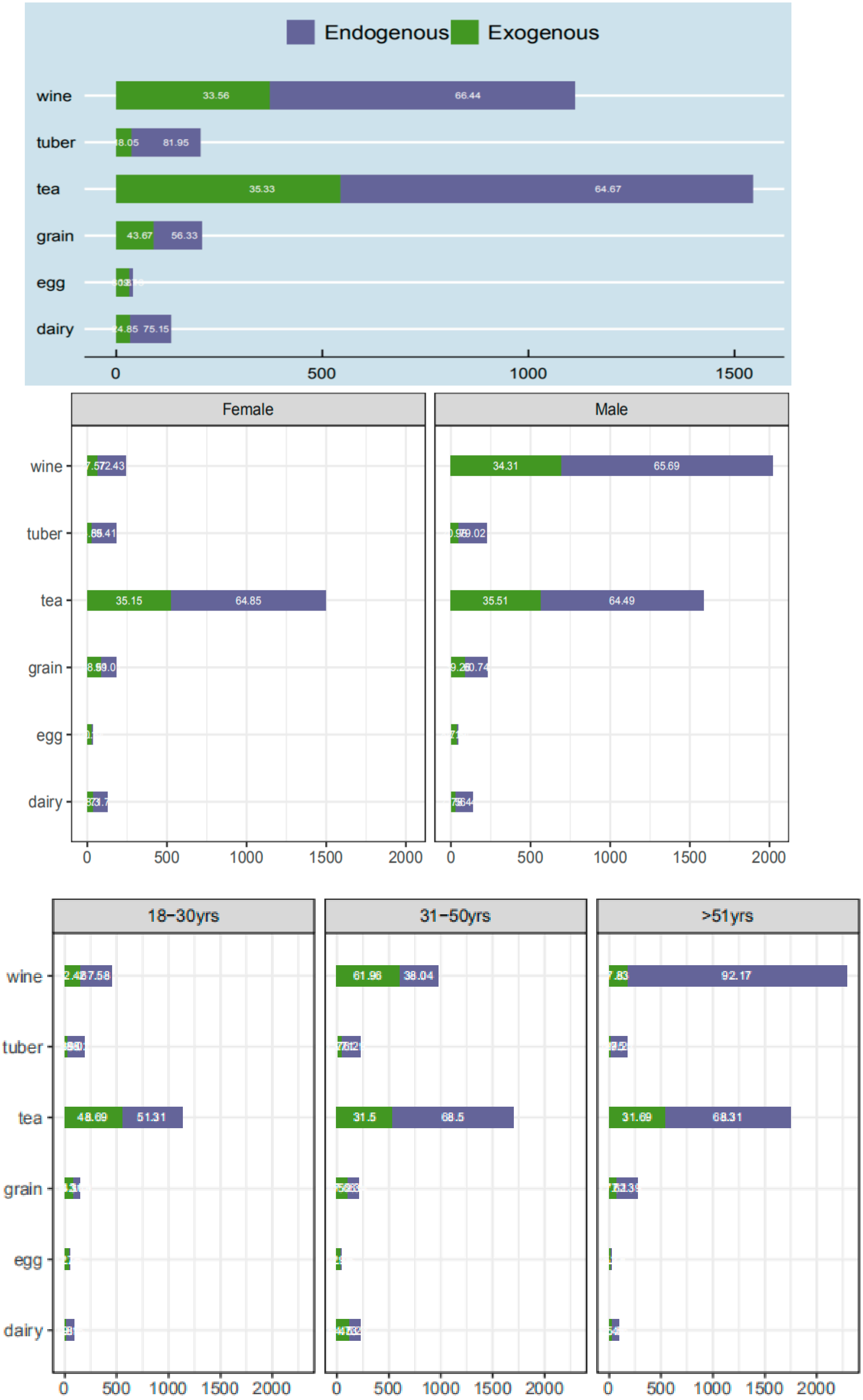

Supplementary Figure S2. The intake of endogenous foods and exogenous foods by Tibetan residents

For supplementary Figure S2:

In order to assess the degree of dependence of local residents on different food

sources-self-produced(endogenous) and purchased(exogenous), in this study, the dietary intakes of each participant were measured from different sources. Local residents in Tibet consume foods produced by themselves2541±632g/d /adult, which significantly exceeds intake of of foods purchased from outside(2281±264g/d /adult)($p<0.05$). 62.9% of local Tibetan residents consumed more endogenous foods than exogenous foods.

In terms of different food categories, the vast majority of the 165 food items investigated are purchased from outside, only cereals, potatoes, dairy products, eggs, cultural-specific tea and wine have two source attributes of both purchased and self-produced. We compared and analyzed the proportion these 6 kinds of food based on homegrown or outsourced consumption, the results showed that except eggs, local residents in Tibet consumed the other five kinds of food mainly from their own production(wine-66.44%, tuber-81.95%, tea-64.67%, grain-56.33%, dairy products-75.15%). From the same food category, males are more dependent on self-produced food than females, and older residents(aged≥51 years) are more dependent on self-produced food than younger residents(aged 18–30 years and aged 31–50 years).
